# Observational studies of treatment effectiveness in neurology

**DOI:** 10.1093/brain/awad278

**Published:** 2023-08-17

**Authors:** Tomas Kalincik, Izanne Roos, Sifat Sharmin

**Affiliations:** CORe, Department of Medicine, University of Melbourne, Melbourne, 3050, VIC, Australia; Neuroimmunology Centre, Department of Neurology, Royal Melbourne Hospital, Melbourne, 3000, VIC, Australia; CORe, Department of Medicine, University of Melbourne, Melbourne, 3050, VIC, Australia; Neuroimmunology Centre, Department of Neurology, Royal Melbourne Hospital, Melbourne, 3000, VIC, Australia; CORe, Department of Medicine, University of Melbourne, Melbourne, 3050, VIC, Australia; Neuroimmunology Centre, Department of Neurology, Royal Melbourne Hospital, Melbourne, 3000, VIC, Australia

**Keywords:** comparative effectiveness, causal inference, propensity score, marginal structural model, methodology, statistics

## Abstract

The capacity and power of data from cohorts, registries and randomized trials to provide answers to contemporary clinical questions in neurology has increased considerably over the past two decades. Novel sophisticated statistical methods are enabling us to harness these data to guide treatment decisions, but their complexity is making appraisal of clinical evidence increasingly demanding.

In this review, we discuss several methodological aspects of contemporary research of treatment effectiveness in observational data in neurology, aimed at academic neurologists and analysts specializing in outcomes research. The review discusses specifics of the sources of observational data and their key features. It focuses on the limitations of observational data and study design, as well as statistical approaches aimed to overcome these limitations. Among the examples of leading clinical themes typically studied with analyses of observational data, the review discusses methodological approaches to comparative treatment effectiveness, development of diagnostic criteria and definitions of clinical outcomes. Finally, this review provides a brief summary of key points that will help clinical audience critically evaluate design and analytical aspects of studies of disease outcomes using observational data.

## Teaser

In this methodological review, Kalincik *et al*. discuss the use of observational studies in clinical neurology, including their limitations and how these can be overcome. They argue that new studies should be designed with a view to the secondary use of their data.

## Introduction

The research using observational data to establish effectiveness of neurological therapies has advanced considerably over the last decade. This is owed to the progress on multiple frontiers—analytical methodology, study design, quality of the available datasets and definition of research questions. During 2022, 57 publications reported studies of non-randomized comparative effectiveness of interventions in neurology, mainly in multiple sclerosis and stroke, but also in epilepsy, Parkinson’s disease, dementia, motor neuron disease, traumatic brain injury, brain tumours and COVID19 (PubMed 29 December 2022, search terms ‘neurology’, ‘comparative’, ‘effectiveness’, ‘therapy’, ‘observational’, excluding ‘meta-analysis’). Our methodological abilities have advanced from simple balancing of covariates in multivariable models, through methods that separate balancing of cohorts from analysis of outcomes, to models of causal inference that estimate causal associations in non-randomized settings. The growing complexity of methods and questions has negatively affected accessibility of the research using observational data to clinical audience. This emphasizes the importance of a general consensus on the key requirements of high quality observational studies.

Complex clinical questions should be answered using complementary approaches and analytical methods, data from diverse sources, and in a broad range of clinical settings.^1^ Convergence of evidence is important and discrepancies among studies should be explored with the aim of elucidating how associations are modulated by specific clinical scenarios.

In this review, we focus on factors that determine analytical approaches to studies of observational data, emphasizing methods for the research of therapies in neurology. We highlight complementarity of randomized and observational studies, discussing the implications of different sources of data and data quality. In exploration of key factors that help determine the choice of statistical methods, we outline the principles of developing a research question and study design. We then discuss different types of bias, with a special focus on treatment indication bias—one of the most prominent limitations of observational data—and its statistical remedies.

This review highlights the key features of contemporary design and statistical methods used in the study of treatment effectiveness in neurology, motivated by examples from clinical neuroimmunology. It builds upon general principles of comparative effectiveness methodology reviewed elsewhere,^2–4^ and is primarily intended for academic neurologists and analysts specializing in outcomes research in neurology, but with general conceptual explanations intended for clinician readers of research papers. For a glossary of key concepts used in the text, refer to Box 1.

Box 1 **Selected analytical concepts in the comparative effectiveness of therapies**

**Table:** 

Average treatment effect (ATE):	Average difference between the effects of compared interventions on the outcome generalized to the entire study population.
Average treatment effect among the treated (ATT):	Average difference between the effects of compared interventions among the patients who received the reference treatment.
Causal contrast	Difference of causal effects of two or more interventions on the studied outcome, estimated within a defined population over a defined time.
Causal inference	A framework that uses specific study designs and estimators to draw causal conclusions based on data.
Counterfactual effects	Estimation of the outcomes in the entire population under a non-observed hypothetical intervention (counterfactual).
Doubly robust estimators	Estimators of effect (usually causal) that combine estimation of treatment allocation with estimation of outcomes to achieve higher robustness to model misspecification.
Directed acyclic graph	A depiction of associations among variables considered in study design, used to determine the best analytical design and strategy to answer the research question.
Generalizability	Degree to which findings of a study apply to the broader context (such as the prevalent population diagnosed with a disease).
Instrumental variable	A variable that independently determines the exposure without influencing the outcome of interest.
Internal validity	The ability of a study to answer research questions without bias.
Misspecification of a statistical model	Incorrect design of a statistical model—including its predictor variables and outcomes—whereby the model does not sufficiently represent the studied reality.
Pairwise censoring	Censoring of entire matched sets rather than of individual participants in a longitudinal study.
Positivity assumption	Assumption of certain statistical models that all patients included in a study had a non-zero probability to be treated with each of the compared interventions.
Propensity score	Probability that an individual is exposed to a certain therapy rather than another therapy, given their set of recorded characteristics.
Pseudo-cohort	Artificially constructed dataset, created from repeated observations from a group of individuals changing over time, often with weighting applied to account for time-varying probability of receiving the intervention(s) of interest.
*P*-value	The probability that results of the same or larger magnitude as the currently observed result would be recorded if the null hypothesis were true.
Target trial	A hypothetical clinical trial that would answer the study question in a randomized setting.
Time-varying confounder	A variable that influences both the probability of exposure/intervention and the outcome, and that changes over time.
Treatment indication bias	Confounding that influences the choice of intervention based on the perceived probability of the outcome of interest.

## Motivation for research of observational data

Randomized controlled trials (RCTs) represent the pinnacle of evidence regarding treatment efficacy. They report the effects of interventions on predefined outcomes within a specific clinical context. Their design is typically optimized to patients who are most likely to benefit from the intervention. To maximize the robustness of the evidence, data in RCTs are generated prospectively, with randomization and blinding to eliminate measured and unmeasured bias, and rigorous data quality control.

Analyses of observational data are now commonly used to complement the evidence from RCTs. This includes themes that typically exceed the capacity, feasibility, funding or ethical constraints of RCTs: development or refinement of diagnostic criteria, comprehensive comparative effectiveness of multiple pairs of therapies, comparative effectiveness of therapies where randomization and blinding are problematic, evaluation of the effectiveness of therapies among patients who are typically excluded form RCTs (e.g. due to age or other medical conditions), evaluation of sequencing of therapies, quantification of long-term effect of therapies, estimation of treatment effectiveness within subgroups of patients and of individual treatment response. Partly due to their capacity to generate evidence in these thematic areas and partly due to their timeliness while relevant RCTs are still underway, high quality analyses of treatment effectiveness in observational data are now considered in clinical decision-making and have influenced diagnostic and treatment guidelines.^5,6^

## Sources of high quality observational data

Two key requisites of any research study are high quality of the research methodology and high quality of the analysed data.

Traditionally, observational data are generated in prospectively designed cohort studies. The advantage of cohort studies is their representativeness of clinical reality (within the constraints of their inclusion criteria), structured follow-up, capacity to integrate non-standard paraclinical methods and predefined research questions.

Similarly, data generated in RCTs can be repurposed to study questions outside of the scope of the original trials. Importantly, this approach invalidates the original randomization as a solution to treatment indication bias (unless the new study question compares outcomes between the originally defined intervention groups, using the original inclusion and censoring rules).^7^ On the other hand, RCTs provide researchers with high quality, harmonized, rigorously acquired and monitored data.

Complementing cohorts and trial datasets, clinical registries are large-scale data repositories, designed to capture information about a disease in conjunction with clinical practice. Their data acquisition is not strictly hypothesis-driven. Cross-sectional registries usually document characteristics of patients diagnosed with defined conditions at a defined time point (e.g. at diagnosis). Longitudinal registries capture information serially, often with variable intervals between data entry points. The complexity of longitudinal registries depends on the studied disease and the breadth of demographic, clinical and paraclinical information recorded. Further, different data types may be acquired at different time points (e.g. MRI and clinical information). This leads to analytical complexities, which can be solved with the use of derived outcomes (such as confirmed disability milestones), interpolation of data-points or simultaneous (multivariate) analysis of multiple outcomes.

The three sources of observational data offer complementary settings for cost-effective outcomes research with ample opportunities for cross-validation.

## Limitations of observational data

Whether a concrete research question is best suited to a study in a cohort or a registry depends on multiple factors. Typically, prospective cohorts are restricted to one or a small number of study sites, with rigorous definition of inclusion, thus favouring internal validity at the expense of generalizability of findings. This is a source of selection bias, which is therefore an inevitable consequence of study inclusion criteria and conditions of enrolment into cohorts. Selection bias should be incorporated into a correct interpretation of results, within the correct clinical context supported by the studied population sample. On the other hand, registries typically operate across a large number of centres and regions, with less strict definition of inclusion, considerable power, less prominent selection bias but with an increased risk of heterogeneity.

In particular, paraclinical information is subject to heterogeneity across different data sources. For example, the widely variable timing, equipment and protocols for imaging of brain or spinal cord in observational datasets remain a significant challenge. While there is presently no generally accepted solution to minimizing this heterogeneity, potential disease-specific solutions rely on definition and universal acceptance of the least common methodological denominators of follow-up, combined with development of robust derived metrics.^8^

Another limitation of retrospective analyses of existing datasets is the restricted availability of required information. Because in most observational studies the research questions are defined after the data have been captured, the design of a dataset, outcomes, metrics and interventions/exposures may not represent the optimal solution to testing the postulated hypothesis.

In retrospective analyses of observational data, data quality may vary among data sources, or even within data sources—among study sites or variables. Therefore, quality of the analysed data requires quantitative evaluation.^9^ Data completeness is assessed as the number/proportion of complete data entry points for essential variables. Syntactic accuracy reflects the number/proportion of variables with values within their plausible range. Consistency corresponds to the number/proportion of data entries that are congruent with other recorded information (such as feasible sequence of events and outcomes). Believability represents the number/proportion of entries that are regarded as true and credible. While believability can be approximated through comparison of the distributions of the recorded variables against their known distributions in the prevalent population (e.g. age at onset of a disease, female-to-male ratio), the ultimate assurance of believability rests with primary source verification. With the exception of the primary source verification, all the above aspects of data quality can be implemented in an automated data quality process.^10^ The results of data quality assessment can be co-reported with main study results or can be used in inclusion/exclusion of study participants (trimming) or their contribution to the analyses (weighting).

## Research question

The key step in generating credible evidence regarding treatment effectiveness is development of a primary research question. It is not uncommon that RCTs and observational studies fail not due to lack of methodological rigour but due to insufficiently developed research question and study design. A good research question is focused, exploring a contemporary clinical or scientific problem, grounded in prior literature and sufficiently specific to be answered in detail. Further, the question should be feasibly answered with the available data, considering their limitations (see above). According to the PICO framework, research questions should specify: (P) problem or population, (I) intervention, (C) comparison and (O) outcomes.^11^

## Study design

The design of all studies of treatment effectiveness includes specification of seven parameters of a target trial: eligibility criteria, intervention(s) and comparator(s), treatment assignment, outcomes, follow-up, causal contrast of interest and statistical analysis.^3^ These are specified prior to patients’ enrolment in RCTs or before data extraction in retrospective analyses of data. Non-randomized studies of treatment effectiveness should aim to emulate a target RCT.

**Figure 1:**
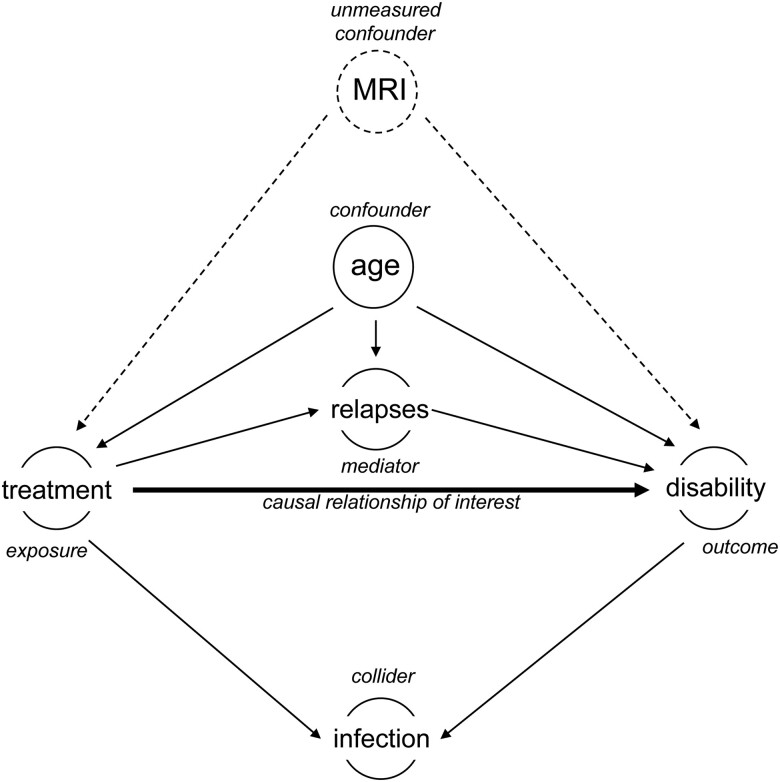
**Directed acyclic graph: an example**. The figure illustrates a causal relationship between treatment and outcome, and relationships with other variables (confounder, unmeasured confounder, mediator of treatment effect and collider) that need to be considered in the design of an observational study and its analysis.

A particularly relevant aid in designing a study of treatment effectiveness is the directed acyclic graph (Fig. 1). Directed acyclic graphs help researchers understand the primary association of interest, its measured confounders, reverse causation, mediators, colliders (using expert input and prior literature) and model hypothetical unmeasured confounding. Based on these expected association and their roles in describing the primary relationship of interest, a statistical model is then designed. A carefully designed model allows the researcher to quantify the primary studied relationship (which may at times be a causal relationship), while accounting for bias but not for other relevant associations (such as mediation of treatment effect).^12^

Design of both prospective and retrospectively analysed studies of treatment effectiveness requires identification of a point of intervention—such as administration (or cessation) of therapy or decision to commence/cease therapy (particularly relevant to the ‘intention-to-treat’ causal contrast), which is then used to define a study baseline. The baseline is the central point in a study design as it enables the separation between events leading into an intervention (that may require balancing or adjustment) and the period over which the outcomes are measured. Certain research questions dictate that a study design considers changes over time to initially assigned intervention (time-varying intervention/exposure). In these designs, a participant may contribute data to different treatment groups at different times. Methods of causal inference suitable for dynamic treatment regimens exist to account for these scenarios.^13^

It is important that the definition of study baseline is equivalent across the compared interventions. Where this is not possible, such as in comparison of treated and untreated states, different definitions of baseline may lead to immortal time bias. This can be eliminated by reconsidering the definition of baseline at the design stage or analytically by modelling treatment as a time-varying exposure.^14^

## Treatment indication bias

The key bias to account for when emulating a target trial comparing effectiveness of treatment strategies is treatment indication bias. This bias is introduced by informed use of therapies in practice—such as treatments that are perceived as more potent may be used more commonly among patients with more severe forms of disease. While in sufficiently powered trials the treatment indication bias at study baseline is accounted for by randomization, non-randomized data require a statistical solution.

The simplest method to balance non-randomized treatment groups at baseline is through matching patients on multiple variables. However, the efficiency of direct matching is low, as it excludes many patients based on information that is not relevant to the outcomes and potentially also treatment allocation (i.e. is not a likely confounder). A more efficient method first identifies variables that are associated with treatment allocation (using a logistic regression model, iterative approaches or even machine learning) and calculates the probability of treatment exposure for each patient based on their characteristics—propensity score. The propensity score is then used to balance the compared groups in several ways—most commonly through propensity score matching or inverse probability of treatment weighting.^15^ Propensity score matching can be viewed as an extreme form of weighting, where only the successfully matched patients are retained in the analysis (with a weight of their contribution to the final model of 1). On the other hand, weighting retains all patients in the analysis, with each patient’s contribution weighted by their probability of being exposed to the reference therapy. Further, inverse probability of treatment weighting can estimate treatment effect in two scenarios (Box 1): the average treatment effect among the treated (ATT) compares the treatment effectiveness in a clinical context in which the reference therapy is typically used. The average treatment effect (ATE) estimates the overall treatment effect among all patients included in the study, irrespective of their actual treatment exposure. The latter provides a result that is more broadly generalizable to the prevalent population. For example, a study of comparative effectiveness showed that natalizumab is superior to fingolimod in preventing relapses of multiple sclerosis when compared only among patients typically treated with natalizumab (ATT) as well as in the entire study population (ATE).^16^ Unlike weighting, propensity score matching allows pairwise censoring, ensuring that equal follow-up times are contributed by matched patients from both groups, thus preserving the balance throughout the follow-up and mitigating attrition bias (see below).

An important advantage of both methods is their ability to separate the balancing of the compared groups from the analysis of treatment effects. Each study should report key demographic, clinical and paraclinical information in the balanced treatment groups and ensure that a sufficient overlap exists between the compared groups before reporting comparisons of the interventions. Where imbalance persists despite accounting for determinants of treatment allocation, the group-balancing method needs to be revised or an additional adjustment is required (such as multivariable adjustment of the model of treatment effect).

Recently, the methods of causal inference have been gaining momentum in neurology. Causal inference aims to establish causal relationships between interventions and outcomes while accounting for measured treatment indication bias. This is of particular importance to questions where strong reverse causation obscures the results of conventional methods. For example, the question of causal effect of pregnancy on the risk of developing multiple sclerosis and attaining significant disability is subject to strong reverse causation, as females without symptoms of multiple sclerosis are more likely to choose to have children. A study of a national registry emulated a target trial using a marginal structural model and a doubly-robust estimator (longitudinal targeted maximum likelihood estimator, see below and Box 1), and found no evidence of an effect of pregnancy on disability in multiple sclerosis, while accounting for time-varying confounders.^17^ Methods such as marginal structural model thus enable adjustment for time-varying confounders throughout the duration of study follow-up.^18^ This is of particular importance in scenarios where persistence on studied therapies is strongly determined by their perceived benefits. Adjustment for time-varying confounding is also relevant to RCTs. For example, an RCT of oestrogen-progestin supplementation compared with placebo in postmenopausal females suggested its association with marginally increased risk of breast cancer. However, this RCT was subject to a significant non-adherence to the active therapy, which resulted in underestimation of the treatment effect. When reanalysed with a marginal structural model accounting for treatment non-adherence, the effect of oestrogen-progestin on the risk of breast cancer was substantially greater.^19^

Causal inference is closely related to the counterfactual framework. Counterfactual analyses compare the effectiveness between therapies over multiple time points, using prediction to estimate the effects of exposures that were not directly observed.^13,18^ The question that these analyses aim to answer is: ‘What if a group of patients were treated with a concrete therapy different to the therapy they were in reality treated with?’ The most common methods are based on G-computation or inverse probability of treatment weighting.

The methods discussed above rely on accurate specification of both the model of treatment exposure and the model of outcome. Doubly robust estimators are a novel class of estimators that combine the two models. These models are more robust to misspecification, as only one of the two models needs to be specified correctly.^20^

The methods of comparative treatment effectiveness assume that no unmeasured confounding affects the analysed data. However, unmeasured confounders are often present, especially in conditions where the pathophysiology is only partially understood. Violation of this assumption can lead to an imbalance between the compared treatment groups, where a true unmeasured confounder would influence both treatment assignation and the outcome and may potentially lead to biased results. In fact, propensity score matching may lead to increase in the imbalance of unmeasured group characteristics.^21^ While the assumption of no unmeasured confounding is not verifiable, robustness of an analysis to a hypothetical unmeasured confounder should be quantified. For instance, Hodges-Lehmann Γ estimates the minimum magnitude of a hypothetical confounder that would be required to change the conclusion of a study. Alternatively, hypothetical latent confounding variables can be included in models of treatment effect.^4^

Positivity is another key assumption of the studies of comparative effectiveness. It requires that all subgroups of the study population can be treated with either of the compared therapies. Its violation may introduce considerable variability in the reported results and a risk of spurious findings.^16^ It is evaluated by comparing characteristics of the treatment groups and accessibility of the study interventions at study sites. Its violations require changes to study design (e.g. trimming of the study population) rather than to statistical methodology.

An approach using instrumental variables is a mirror opposite of propensity score-based methods.^22^ Rather than adjusting analyses for observed confounding of the relationship between the exposure and the outcome, instrumental variable serves as independent determinant of exposure. Its association with the studied outcome must be mediated solely through the exposure/intervention, which it determines. However, true instrumental variables only determining the choice of treatment but not modifying the outcome are rare in neurology.

## Other sources of bias

Among other sources of bias to be considered in both RCTs and analyses of observational data is the selection bias. It is introduced by restriction of patient inclusion. Selection of patients reduces generalizability of results of research studies but is a necessary step to ensure their internal validity. Depending on the primary research question, a researcher may choose to prioritize internal validity over generalizability or vice versa.

Detection bias results from differences in standard follow-up used in different therapies. It may influence the likelihood of detecting a study outcome and is mitigated by the use of secondary outcomes that are robust to follow-up variability or adjustment of analyses for follow-up protocols.

Informative censoring or attrition bias may affect the results of studies in which considerable differences exist in treatment adherence or persistence. Patients with therapies that are perceived as being associated with study outcome may be censored before the outcome is reached. This problem can be remediated by intention-to-treat causal contrast (which, in observational settings, assigns a patient to a treatment group based on the therapy initiated at/after study baseline irrespective of any later changes to this treatment), accounting for determinants of treatment discontinuation, or pairwise censoring.^19^

## Common themes in studies of treatment effectiveness

### Comparative treatment effectiveness and individual treatment response

In many clinical scenarios when randomization and blinding are not feasible, high-quality observational studies represent a viable alternative to RCTs. For example, a propensity score-matched study in major depressive disorder showed that the use of anxiolytic medication is lower after electroconvulsive therapy versus pharmacotherapy only.^23^ Appropriately designed studies of observational data complement RCTs and improve their generalizability to prevalent populations, within the constraints of the limitations discussed above. For example, a propensity score-matched and inverse probability of treatment weighted cohort study showed that endovascular treatment is not superior to medical management in treatment of cerebral venous thrombosis in a representative cohort from four countries.^24^ Hence, studies conducted in treated prevalent populations, such as prospective cohorts or national/international registries, are typically directly translatable to the target populations, and may even inform off-label use of therapies. For instance, analyses of observational data have demonstrated that rituximab, used off-label in multiple sclerosis, is highly effective in preventing relapses of multiple sclerosis when compared to moderate-efficacy disease-modifying therapies^25,26^—an observation that was recently confirmed in an RCT.^27^

The importance of the clinical context of an intervention has been highlighted in a series of studies that compared two moderate-efficacy immunotherapies for multiple sclerosis. The studies showed that among patients with previously well controlled disease, the effectiveness of dimethyl fumarate and teriflunomide in preventing relapses is similar. However, among treatment-naïve patients or those with recently active disease, the effectiveness of dimethyl fumarate is slightly superior.^28–30^

The question of clinical context is often explored through subgroup analyses in RCTs. Contingent on the available power, a similar approach can be applied in observational data. In fact, inclusion criteria, which are typically broader in observational studies than in RCTs, allow for more comprehensive study of subgroup effects, including underserved patient groups. For instance, patients with multiple sclerosis older than 55 years are typically excluded from RCTs, which prevents evaluation of treatment effectiveness among the elderly.

**Figure 2:**
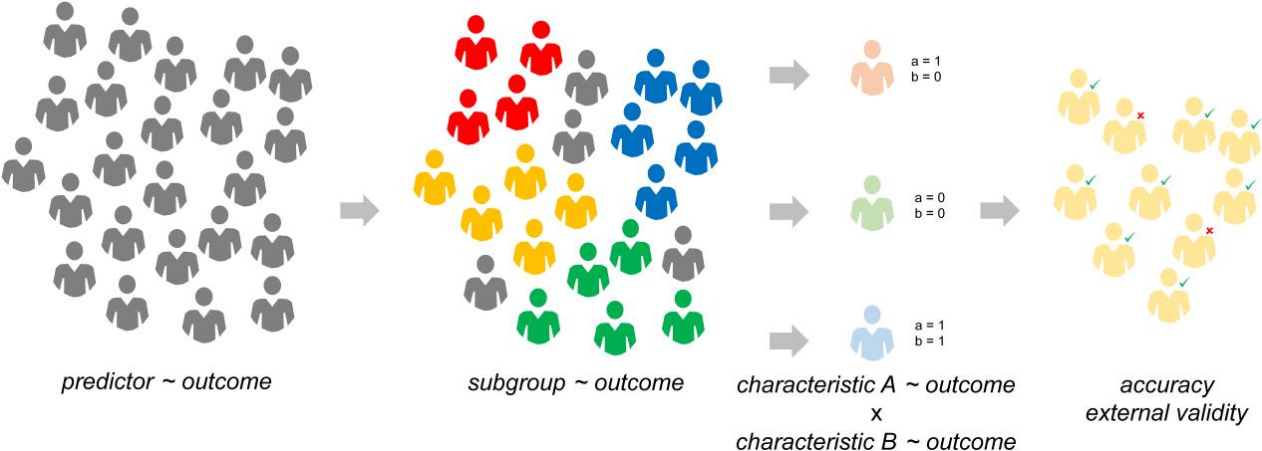
**From group effects to individual outcomes**. Conventional predictive models of outcomes first establish general associations between predictors/exposures and outcomes at the level of populations (*left*). The outcomes in discrete subgroups (corresponding to different clinical scenarios or demographic strata) are then studied (*middle left*). It is advantageous to stratify the population based on the relevant predictors/exposures identified in the whole population. To establish discriminative ability of the predictive markers, the individual predictors, their sums and their interactions are studied in patients, accounting for their individual constitution of relevant characteristics (here depicted as ‘a’ and ‘b’; *middle right*). Accuracy and external validity of the resulting predictive models is established by testing the models in individuals from a non-overlapping (validation) cohort (*right*).

Detailed understanding of the effectiveness of interventions within subgroups helps elucidate clinical modifiers of treatment effect and may lead to hypotheses about their underlying biological mechanisms. It also provides clinicians with immediately translatable information. For example, the superiority of natalizumab over fingolimod in preventing relapses of multiple sclerosis is most pronounced among younger patients, females and those with prior history of relapses.^31^ Even though effectiveness within subgroups and individuals are not synonymous, sequences of appropriate analyses may translate subgroup effectiveness into estimation of individual treatment response (Fig. 2). For instance, pretreatment frequency of multiple sclerosis relapses co-determines the magnitude of the effect of disease-modifying therapy on relapse frequency among individuals with relapsing but not with progressive multiple sclerosis.^32^ This phenomenon is apparent from the differential correlation between the individual pre- and on-treatment relapse frequencies within the two strata—relapsing and progressive multiple sclerosis. Future benefit of switching to a second-line multiple sclerosis therapy can be estimated by modelling the interactions of several patient characteristics—disability, age, relapse frequency and enhancing cerebral lesions—with the magnitude of treatment effect.^33^ Alternatively, a different approach first estimates the associations of patients’ demographic, clinical and paraclinical characteristics with the magnitudes of effects of two studied interventions within two sets of available RCTs.^34^ Differences in the magnitudes of these associations are then used as a set of weights applied to individual’s characteristics. The sum of these weighted characteristics calculated for each patient is then used to predict the difference in the effects of the two compared therapies in the given individual.^35,36^ This approach represents an example of an evolution from understanding average treatment effect in a population, through effectiveness within subgroups, to estimation of individual treatment response (Fig. 2).

However, associations between predictors and outcomes are not necessarily constant across different strata of treated populations and simplification of their relationships to their linear combination may lead to variability of error in different patient subgroups. To circumvent this problem, modelling of higher dimensional interactions is required. The ultimate realization of a high dimensional predictive algorithm, which allows non-linear interactions among predictors, is enabled by machine learning approaches. Examples of their application are forecasting of seizures in epilepsy or prediction of response to anti-seizure medication.^37,38^ In addition to allowing high dimensional interactions among predictors, machine learning also iteratively optimizes the accuracy of the predictive model. In chronic neurological conditions, prediction of trajectories (outcomes evolving over time) based on data captured in clinical practice at irregular intervals requires new analytical solutions.^39^ The key clinical limitations of machine learning algorithms are the lack of understanding of individual predictors, limited interpretability of their high dimensional constructs and limited generalizability beyond the learning cohort. To facilitate their translation into practice, they should be presented alongside the individual, clinically accessible predictors. Fortunately, novel approaches can be used to estimate contribution of each variable to the final prediction are finding their way into analyses of clinical data. These methods can be combined with standard machine learning algorithms, such as gradient boosting methods. An example was recently provided in a study that used Shapley additive explanations (SHAP), which compute the contribution of each variable to the prediction of outcome in each individual using cooperative game theory.^40^ This example identified treatment duration, disability and MRI features as predictors of reactivation of multiple sclerosis observed within 3–6 months in individuals participating in the RCTs of cladribine.

To establish generalizability of predictive models, one needs to demonstrate their performance in a distinct study populations through validation. For any model to be truly predictive, the study periods during which the potential predictors and the predicted outcomes are recorded need to be non-overlapping. An illustration is provided by the CHA_2_DS_2_VASc score, which estimates an individual risk of future thromboembolic event while patients are diagnosed with non-valvular atrial fibrillation.^41^

**Figure 3:**
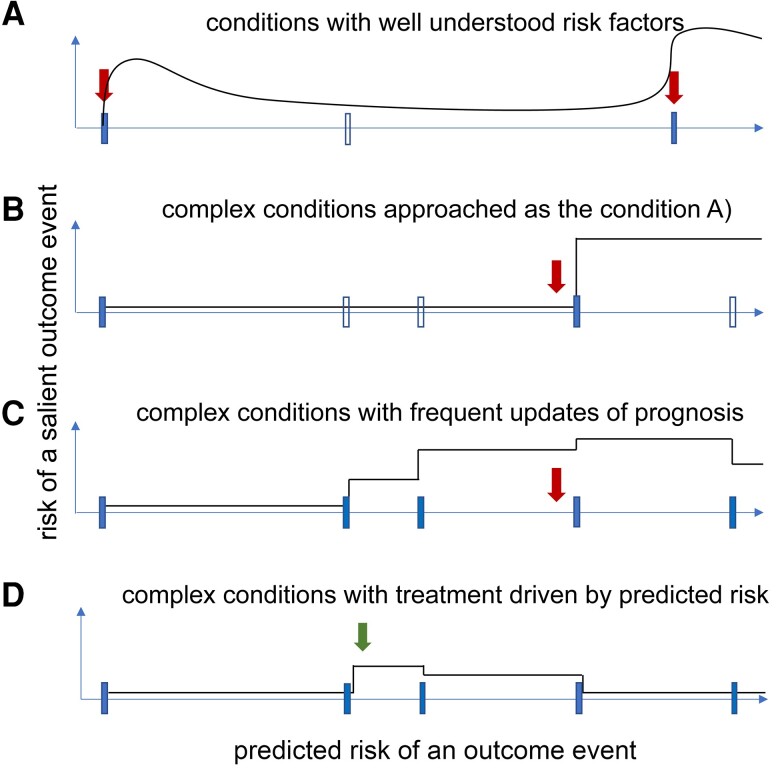
**Prediction of long-term outcomes: a clinical perspective**. In conditions with well understood causal relationships such as stroke (**A**), reliable long-term prognostic models of adverse disease episodes (red arrows) are plausible. These are typically only updated upon change in clinical circumstances. In amnesic conditions such as multiple sclerosis (**B**–**D**), predictions of long-term outcomes conducted at a single time point and not updated between adverse disease episodes become increasingly inaccurate over time (**B**). In this example, the patients’ risk is not updated until the clinical assessment following the next event (blue marker). However, in practice, a clinician would intuitively update the predicted prognosis at every assessment (blue markers), combining the prior prediction with new information (**C**). This enables the clinician to respond to change in the predicted risk by modifying therapy (green arrow) and potentially preventing the adverse outcome (**D**). Black lines = predicted risk; white markers = clinical assessments; blue markers = clinical assessment with update of prediction; red arrows = exacerbations/adverse outcomes; green arrow = change of treatment.

To offer their patients honest, truly individualized prediction, clinicians must be provided with the margin of uncertainty surrounding each prediction. To this end, Bayesian approaches offer two considerable advantages: similar to clinical reasoning, they estimate posterior probability as an update of a prior probability based on new (individual) data. This is particularly useful in diseases whose long-term course is partly amnesic to their prior clinical history—such as multiple sclerosis, myasthenia gravis and some epilepsies. In these conditions, a prediction of long-term outcomes consists of a series of shorter-term predictions updated at the time of clinical reviews (Fig. 3). Bayesian prediction also enables estimation of individual 95% prediction intervals to quantify accuracy of prediction in an individual. Another important feature of predictive models is their ability to accurately classify patients who will experience different outcomes (discriminative ability).

### Diagnostic criteria and outcomes

Accurate definitions of diagnoses and outcomes, which closely reflect clinical reasoning in diagnostics and prognostics, are key requisites of clinical studies whose results are relevant and translatable into practice. Studies of observational data have played central roles in refining formal definitions of diagnoses and clinical outcomes.

In particular, eligibility criteria of randomized and observational studies of treatment effectiveness are built on diagnostic criteria. The process of developing criteria that objectively establish a diagnosis with acceptable certainty is typically iterative. For example, according to the 2017 McDonald diagnostic criteria for multiple sclerosis, intrathecal synthesis of oligoclonal antibodies can now replace the criterion of dissemination in time, based on its highly accurate prediction of the definite diagnosis.^42,43^ Recently, motivated by clinical and laboratory findings, a more substantial revision of the traditional diagnostic classification has proposed a move away from the phenomenological categories (relapsing and progressive multiple sclerosis) in favour of quantitative assessment of the underlying pathogenesis—focal inflammation and neuronal loss.^44^ Another illustration is provided by Rasmussen encephalitis, where the classical triad enables a definitive diagnosis. However, the clinical picture is often incomplete early in the disease. Progressive nature of the changes and/or tissue diagnosis eventually leads to the correct diagnosis.^45^ In general, early prognostic markers, when incorporated into diagnostic criteria, can enable earlier and more accurate diagnosis.^43^ In many areas of medical diagnostics, supervised machine learning is being explored to optimize early diagnosis, with close attention to external validity. Optimized diagnostics, especially of phenotypically minimally expressed conditions, can help limit the heterogeneity among patients enrolled in future RCTs.

Another essential aspect of studies of treatment effects are objectively defined clinical outcomes. While in many neurological diseases modified Rankin Scale provides sufficient discrimination, in conditions with complex disability patterns, more comprehensive scales are required. These scales are typically derived from primary observations (such as neurological examination), combining measurable aspects of the function of the nervous system, with the aim to quantify the full breadth of a disease. Clinical outcomes are typically defined and refined using repurposed data from RCTs, cohorts or registries—that is in observational settings. Their performance criteria consist of reliability, sensitivity, validity, efficiency and acceptability.^46^ Diseases such as Alzheimer’s dementia, myasthenia gravis, multiple sclerosis and others provide many examples.^47–50^

### Other research questions

So far, most studies evaluating effectiveness of therapies in neurology compared two or three active interventions. Comparisons of active therapies to no treatment in observational data remain problematic. This is due to the usual lack of overlap between groups who received therapy and those who remained untreated in scenarios where effective treatment options are available. In these instances, balancing of groups at baseline would not eliminate the treatment indication bias. The counterfactual framework may represent a viable solution, allowing estimation of the outcomes in hypothetical (pseudo)cohorts during time exposed to versus not exposed to therapy.^51^ While this solution would still not enable a comparison of truly untreated versus treated patients, it allows a balanced comparison of outcomes among patients who all fulfilled the criterion of eligibility for treatment (e.g. demonstrated by being ever exposed to a therapy), comparing their untreated versus treated periods of follow-up. Besides comparisons of treatment effectiveness, observational data are also being used to explore management of treatment failure, treatment sequencing or timing of commencing, escalation or discontinuing therapy.

Validation of findings of research studies, whether randomized or otherwise, remains at the heart of the scientific method. A *P*-value alone does not warrant that an estimate of a studied association is accurate or even correct. An external validation of results by independent researchers in a non-overlapping dataset provides findings of studies with substantial additional gravitas. Increasingly, validation studies have been presented together with main analyses, either using truly independent external datasets or creating discovery and validation datasets by randomly dividing study populations. External validation is of particular importance in predictive studies, where it is essential for quantification of their accuracy and clinical application.^52^ Where a validation analysis does not replicate the discovery analysis, this does not necessarily invalidate the prior research. On the contrary, it provides researchers with an opportunity to study the modelled error terms, residuals and explore differential associations conditional on different clinical scenarios and patient characteristics. Through this exploration, validation can help refine the results of studies and provide more detailed understanding of underlying clinical contexts.

Finally, clinicians and researchers should be discouraged from over-reliance on *P*-value, which is a frequentist assessment of the strength of evidence provided by an analysis. Because a *P*-value is conditional on the available power and does not enable one to directly reject an alternative hypothesis, it needs to be interpreted with caution. From this point of view, Bayesian approach may provide a more intuitive and symmetric interpretation of the results of statistical analyses. Most importantly, it is the magnitude of the reported effect (such as a difference in the effectiveness between two interventions) and its distribution that provides the most relevant perspective of a reported result.

## Conclusion

RCTs, cohorts and registries provide researchers with a wealth of data. With application of appropriate study design and analytical methodology, these data are being transformed into new evidence that complements the original findings of RCTs and pushes the boundaries of evidence-based therapy, diagnosis and prognostics in neurology. Contemporary global guidelines in neurology increasingly incorporate findings from credible analyses of observational data, facilitating their translation into clinical practice and improving the effectiveness of disease management. It is important that future randomized and non-randomized studies are designed with the view of secondary use of their data, in order to support this important endeavour. Thanks to advances in statistical methodology, the research questions that can now be addressed include not only comparisons of effectiveness of therapies, but also timing of their use, discontinuation, prognostics and individual treatment response. While the responsibility for the quality of reported research rests primarily with researchers, reviewers and journal editors, translation of knowledge into practice and further research is carried by the broader clinical-scientific community. We hope that this methodological overview will help the readers navigate the rapidly expanding landscape of studies of treatment effectiveness in neurology. We have summarized the key methodological features that a reader should critically appraise in publications of studies of observational data (Box 2).

Box 2 **Key attributes of reports of analyses of observational data**

**Table:** 

Data source	Were the analysed data generated by a cohort study, recorded in a registry or repurposed from a randomized controlled trial?
Eligibility criteria	Are the eligibility criteria clearly defined? Is the included population consistent with the target population and the research question? Were any clinically relevant groups excluded from the study?
Treatment strategies	Are the compared treatment strategies clearly defined? Is confounding by relevant prior or concomitant therapies considered and accounted for?
Treatment indication bias	Are all statistical methods reported clearly? What methods were used to mitigate treatment indication bias? Does the report list all variables used in calculation of propensity scores, weights and adjustment? Has the study design considered heterogeneity of the data (e.g. across sites, geographic areas or over time)? Are the results of models of treatment exposure reported (e.g. logistic regression of the propensity score or similar)?
Other bias	What methods were used to account for detection, attrition and immortal time bias? Was unmeasured bias considered?
Outcomes	Are the study outcomes clearly defined and relevant to the research question? Is the follow-up and censoring described and accounted for?
Reporting of results	Do the results not overinterpret *P*-values? (Is the lack of statistical difference interpreted as lack of evidence for a difference rather than an absence of difference? Are the results presented in context of the available power?) Are results presented as point estimates (e.g. mean) and interval estimates (e.g. 95% confidence interval)? Are standardized mean differences (rather than *P*-values) used to compare patient characteristics at baseline?
Interpretation	Is magnitude of effect interpreted separately from the statistical significance? Are limitations of the study sufficiently recognised? Are the results interpreted within the constraints of the study selection bias? Are the conclusions of the study in keeping with the results and its limitations?

Research of therapies in neurology has entered an exciting era when the available data can answer highly relevant and complex clinical questions, beyond the scope of their original studies. Together with the growing volume of information, the increasingly more sophisticated methodology is allowing us to refine treatment of neurological diseases at a scale not seen before.
